# Controlling electricity storage to balance electricity costs and greenhouse gas emissions in buildings

**DOI:** 10.1186/s42162-022-00216-5

**Published:** 2022-07-27

**Authors:** Vahid Aryai, Mark Goldsworthy

**Affiliations:** Commonwealth Scientific and Industrial Research Organisation, 10 Murray Dwyer Cr., Mayfield West, NSW Australia

**Keywords:** Carbon intensity, Net zero emissions, Carbon cost, Electricity tariffs, National Electricity Market, MPC

## Abstract

The optimal management of flexible loads and generation sources such as battery storage systems in buildings is often concerned with minimizing electricity costs. There is an increasing need to managed flexible resources in a way that minimises both costs and carbon emissions. Minimising emissions of grid consumed electricity requires quantification of the carbon emissions intensity of the electricity grid, so first we develop a real-time emission intensity model of the Australian National Energy Market using a power-flow tracing approach. This model reveals that electricity price signals currently do not drive consumers toward using electricity at times of lower emissions. For example, the mean and peak emissions intensity during low electricity tariff periods are the same or slightly higher than those during high tariff periods, while the 30-min wholesale electricity price in each region has no significant correlation with the emissions intensity of electricity consumed in that region. The emissions model is then used to investigate the extent to which controlling a battery storage system to minimise costs under existing electricity tariff structures also leads to minimisation of greenhouse gas emissions for a case study commercial office building. Results show that reducing emissions does indeed come at the expense of increasing costs. For example, annual operating cost savings reduced from 31% to 20% when the battery control was changed from minimising costs to minimising emissions. This has important implications for buildings seeking to reduce emissions as well as for the design of electricity tariffs.

## Introduction

Many countries are dealing with the negative impacts of climate change driven by rising greenhouse gases (GHG) emissions. In Australia, electricity production accounts for 33.6% of the total GHG emissions (CSIRO 2021). A major contributor to these emissions is electricity production, of which 74% is derived from burning coal and gas (AEMO 2022c). The proportion of coal-based electricity production in other developed countries is very close to Australia’s with 75%, 74% and 71% for the USA, China and India, respectively (Mbungu et al. 2020). Commercial buildings are among the key users of this electricity consuming approximately 25% of the electricity on the National Electricity Market (NEM) (Energy 2022). However, there is also a rising penetration of renewable generators, and the relative timing of their output means that the effective emissions intensity of generated electricity varies over the course of the day and from day to day.

The variation of this emissions intensity has important implications for end consumers seeking to reduce their emissions or reach Net Zero Emissions (NZE) targets, and also for ensuring that investment in renewable generation achieves maximum benefit by offsetting the highest emission generation (Lu et al. 2021). Many building operators and companies are interested in reducing not only their total electricity costs, but also their emissions (Mbungu et al. 2020).

The extent to which minimising real-time emissions coincides with minimising the cost of electricity purchased from grid under typical electricity tariffs is an open question especially while carbon prices remain relatively small compared to the overall electricity cost seen by the consumer. For example, although Australian’s de-facto carbon price (the ACCU) increased by 180% in 2021 (Fowler 2022), if it were charged directly to consumers it would still represent < 15% of a typical monthly electricity bill. Hence minimising electricity bill costs, even where carbon prices are included, does not necessarily lead to minimising carbon emissions.

In this work, we consider the question; “Does using a battery storage system to minimise electricity costs under existing tariff structures at a consumer site lead to minimising of emissions?”. To answer this, we first apply power flow tracing methods to calculate real-time consumption-based emissions intensity of the Australian National Electricity Market using the real-time generators’ supervisory control and data acquisition system (SCADA) data, and the interconnector flow and regional demand data provided by the Australian Energy Management Operator (AEMO). We then use these estimates combined with electricity spot price and tariff data from the utility provider in a commercial building case study where a battery storage system is controlled to minimise costs and emissions with varying weighting factor. A range of photovoltaic-battery systems are modelled, and a model predictive control (MPC) algorithm used to control the battery. Cost and emissions reduction for the site are explored along with the impact of parameters including increasing carbon price and reducing capital costs.

## Previous work

Photovoltaic (PV) cells and battery storage systems are widely used for energy management in microgrids and commercial buildings (Antoniadou-Plytaria et al. 2019; Sepúlveda-Mora and Hegedus 2021; Mariaud et al. 2017). Typically these systems are used for minimising the cost of electricity via peak load shaving and energy arbitrage, sometimes in addition to providing reliability and backup functions. Recently Riekstin et al*.* (2018) considered control of PV-battery systems to minimise emissions ignoring costs, while a few studies have considered the problem of minimising both emissions and costs. For example, Nojavan et al*.* (2017) employed ε-constraint method and fuzzy-based selection of the optimal solutions for optimizing a hybrid system of PV-battery-fuel cell in terms of emission and cost. The authors applied their proposed methodology to a case study and found that a simultaneous reduction in total costs and emissions was achievable. However, their work considered a single average grid intensity factor for emissions estimation (Ren et al. 2016).

Most research on controlling battery storage systems using MPC focuses on cost-saving and peak load management as opposed to emissions reduction. Examples of the applications of stand-alone battery storage systems for cost saving and peak load reduction can be found in the works of Vedullapalli et al*.* (2018) and Elmouatamid et al*.* (2019). Vedullapalli et al*.* (2018) utilised a two-stage load forecasting approach, for short- and long-term trends and MPC for load management of a university building. The authors reported 13.5% annual savings. Elmouatamid et al*.* (2019) minimised the grid import in a simulated big-data centre employing a battery storage system controlled by an MPC strategy and showed that controlling the state of charge of the battery by the MPC strategy could successfully decrease the import from the grid.

Several authors accounted for the gradual decrease in emissions intensity over time, for example due to commissioning of renewable energy projects. Allouhi (2020) used a genetic algorithm for optimizing PV design capacity based on a levelised cost of electricity function and a cumulative environmental benefit function (accounting for emissions). To estimate the latter, the authors considered a linear regression model of the grid emission intensity factor. Mariaud et al*.* (2017) used a mixed-integer linear programming approach to optimise the technology selection, capacity and operation of PV and battery systems for a distribution centre. They found that the optimised PV and battery system can reduce the import from the grid and carbon emission by 30% and 26% respectively, in exchange for a possible increase in overall costs. To account for the time-variances of the emission intensity factor of the grid, they assumed the emissions intensity factor as a step function with 1.5% yearly reductions.

In reality the emissions intensity of grid consumed electricity varies hour-to-hour over any given day, and hence the emissions reduction from displacing grid electricity through on-site PV generation or battery charging/discharging also varies over similiar time-scales.

Several researchers considered fitting a linear function for the hourly change in total CO_2_ emissions (∆E) against the hourly change in total network demand (∆D), in order to obtain marginal emission intensity factors (MEF) as ∆E/∆D (McKenna et al. 2017; Sun et al. 2019). This method was originally proposed by Hawkes (2010) and has been modified by several researchers (McKenna et al. 2017; Sun et al. 2019; Siler-Evans et al. 2012). Sun et al*.* (2019) employed this emission intensity modelling technique in conjunction with an emission arbitrage algorithm for studying the potential of PV-battery systems for emissions reduction. They concluded that the battery could fully repay its CO_2_ costs of manufacturing if it were controlled to minimise operational emissions.

The main issue with the Hawkes’ model and its modifications is that they do not consider the power flows between different regions of the electricity network where different combinations of generator types (with different emissions intensity factors) are in use. This issue is also present for the other MEF, and grid mix emission factor models introduced above. Moreover, the low temporal resolution of the linear and step models does not allow for time of use (TOU) optimisation of PV-battery operations. This was shown by Kopsakangas-Savolainen et al*.* (2017), who employed an emission intensity model with daily variations (hourly resolution) in a TOU optimisation and demonstrated a 3–8% emission reduction in a building based on the Finnish network. This implies the importance of developing real-time emission intensity models with high temporal resolutions.

Recently power flow-tracing methods Bialek (1996) have been applied to map electricity flows and emissions in real-time from the original generation source to the point of consumption for the EU and US markets (Tranberg et al. 2019; Chalendar et al. 2019). Tranberg et al*.* (2019) applied flow-tracing to compare the consumption-based and production-based carbon emissions of the European electricity market. They showed that there is a significant difference between the *consumption*-based emissions (the emissions associated with a unit of energy consumed at a given point on the network) and the *production*-based emissions (the emissions associated with a unit of energy generated within a given area of the network), particularly for countries with an imbalanced distribution of renewable to non-renewable generators. They developed a real-time framework and online mapping service (Map 2022). The same methodology has been applied to the US market by de Chalendar et al*.* (2019) who also discuss the importance of considering the emission intensity of the source and cross-border (inter-regional) flows. The available toolboxes for mapping emissions in Australia include a native platform (NEM 2022) which illustrates overall emissions of the country and the recently updated consumption-based online platform developed by Tranberg et al*.* (2019). These platforms, however, do not allow with a detailed analysis of the spatial and temporal varying emission intensity of NEM.

## Significance of the research

To our knowledge, consumption-based emission modelling of the Australian NEM has not been described in the literature. The online platform developed by Tranberg et al*.* (2019) provides limited information regarding the source of electricity being used in each NEM region. The NEM is a particularly interesting case study since it consists of regions and time-periods where there is a very high penetration of renewable generation, coupled to a geographically diverse network with limited inter-connections between regions. For example between February 2021 and 2022, production was more than 99% renewable in Tasmania while in the neighbouring region of Victoria, 71% of electricity was produced from burning brown coal (AEMO 2022c). The NEM is also operated as an open market where electricity is traded over short timescales in response to fluctuating price signals. As such, analysis of this network can provide insights into how other networks around the world may operate in the future, particularly as renewable generation increases. This work informs consumers in different NEM regions on the emissions intensity of their grid consumed electricity allowing them to implement, for example, strategies to reduce emissions such as load shifting and load shedding.

This work extends the previous research on optimisation of PV-battery systems under different network tariffs (Bloch et al. 2019; Young et al. 2019; Parra and Patel 2016) to the case of both emissions and cost minimisation. Analysis of the time-correlation between different network tariffs and the real-time carbon emissions intensity can help the electricity market operator, regulators and energy companies to revise tariffs to better align them with emissions reduction targets. Additionally, the consumption-based emission intensity model reveals the spatial and temporal evolution of the emissions within different regions of NEM which could be used to guide planning of new generation installations. This capability cannot be realised via the existing production-based emission intensity models.

The organisation of the remainder of this paper is as follows: Sect. 4 provides the methodology of the research. It describes the power tracing model for estimating consumption-based emissions on the NEM and presents the methodology used in the PV-battery system analysis. Section 5 analyses the time-dependent emissions intensity on the NEM in comparison to network and wholesale-based tariffs. Results from the commercial building case study are described in Sect. 6. Finally, Sects. 7 and 8 provide discussions on the limitations of this research, recommendations for future work and conclusions.

## Methodology

### Overview

**Fig. 1:**
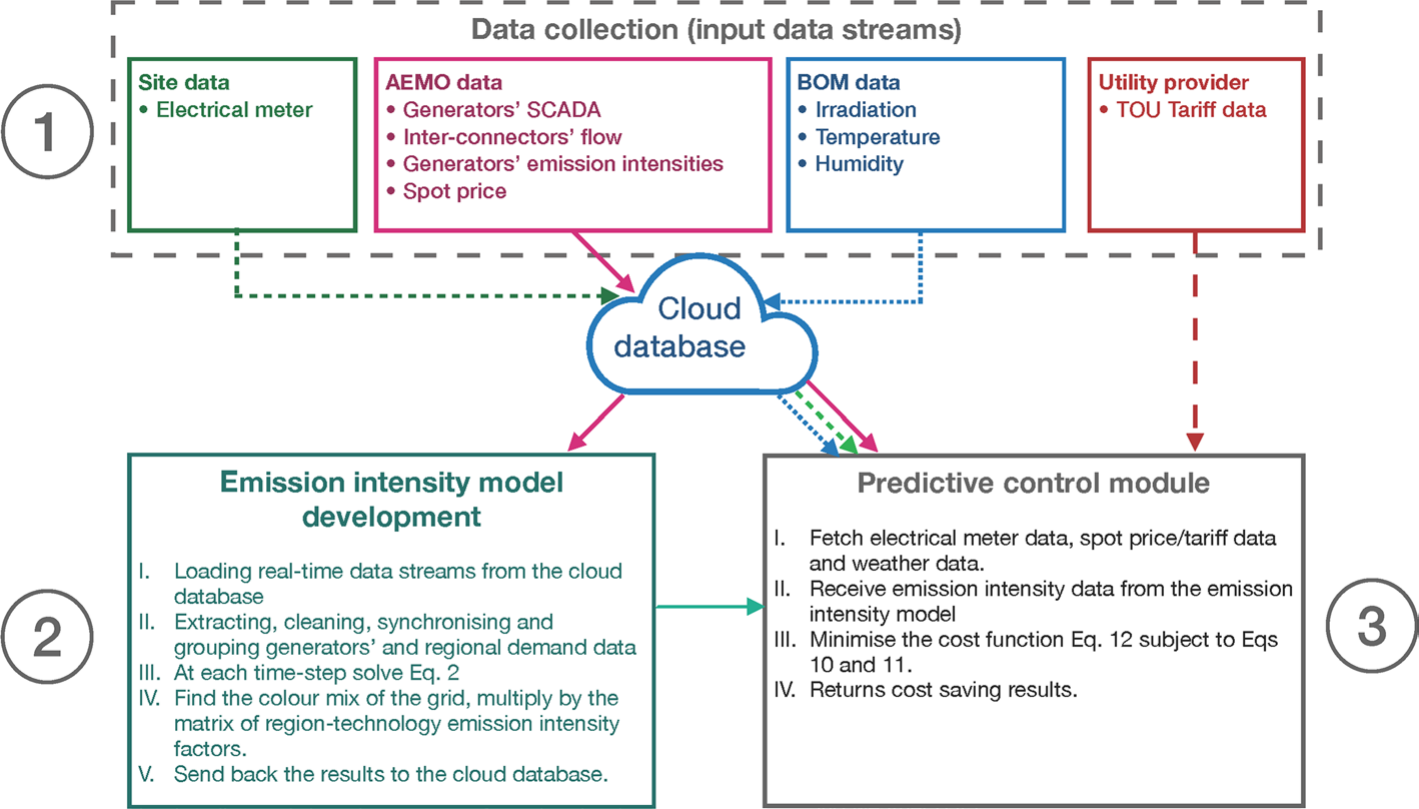
Schematic of the analysis framework, (1) data collection, (2) consumption-based emission intensity model, (3) MPC module for obtaining the cost-saving and emissions reduction results

This section introduces the overall methodology of the research for developing a consumption-based emission model of NEM and the utilisation of this model in a model predictive control (MPC) framework. A schematic of the modelling framework is shown in Fig. 1.

Historical site electricity consumption and gird emission intensity data were fed to a scheduler for simulated control of a battery charge/discharge actions to minimise bill and emission costs. The proposed scheduler is based on an MPC concept, also known as Receding Horizon Control. Through this algorithm the scheduler receives the current and future states of the battery and the modelled PV generation and determines the optimal solution that minimises an objective function of the microgrid’s electricity and emission costs over a prediction horizon. After obtaining the best solution, the first control decision is selected. These steps are repeated until the end of the horizon.

In this modelled scenario evaluation framework, even though the complete historical data is available, at each time-step, only data two days ahead (the prediction horizon) was provided to the scheduler. This allows for updating the demand charge due to the battery charging solution in a realistic way (without future knowledge). The following objectives were investigated:

1. The relative potential for electricity and emission costs saving using a PV-battery system.
2. The influence of time of use versus wholesale-based tariffs on the cost and emissions saving.
3. The sensitivity of the savings to increasing carbon price.

### Emissions intensity model

The flow-tracing method as described by Tranberg et al*.* (2019) is employed herein for modelling the NEM’s consumption-based emission intensity. The basis of the flow-tracing method is the principle of proportional sharing, the details of which can be found in the work of Hörsch et al*.* (2018). The flow-tracing approach considers the rule of conservation of electricity within a network, so that at each time-step the inflow to each region of the network should equal the outflow. This can be expressed as:

1 $${Load}_{i}+\sum_{k}{Flow}_{i\to k}=\sum_{tech}{Generation}_{i,tech}+\sum_{j}{Flow}_{j\to i},$$

where ${Load}_{i}$ is the operational demand in region *i,* ${Flow}_{i\to k}$ is the outflow to the neighbouring regions from region *i*,${Flow}_{j\to i}$ is the inflow from neighbouring regions to region *i,* and ${Generation}_{i,tech}$ is the power generated by a given end-use technology. By introducing the ‘colour mix’ matrix ${Q}_{j,tech}$, which is the proportion of electricity generated by different technologies in each region, into Eq. (1), and re-arranging one can obtain the following Eq. (2):

2 $$\sum_{j}\left\{\left({Load}_{j}+{Flow}_{j\to k}\right).{\delta }_{i,j}-{Flow}_{j\to i}\right\}.{Q}_{j,tech}={Generation}_{i,tech},$$

where ${\delta }_{i,j}$ is the Kronecker delta. By solving the linear system in Eq. (2) for all the regions at each time-step, the colour mix matrix can be obtained. Multiplying ${Q}_{j,tech}$ by a matrix of carbon intensity factors of the corresponding region-technology pairs and summing the results for each region gives the consumption-based emission intensity of the grid for each region.

**Fig. 2:**
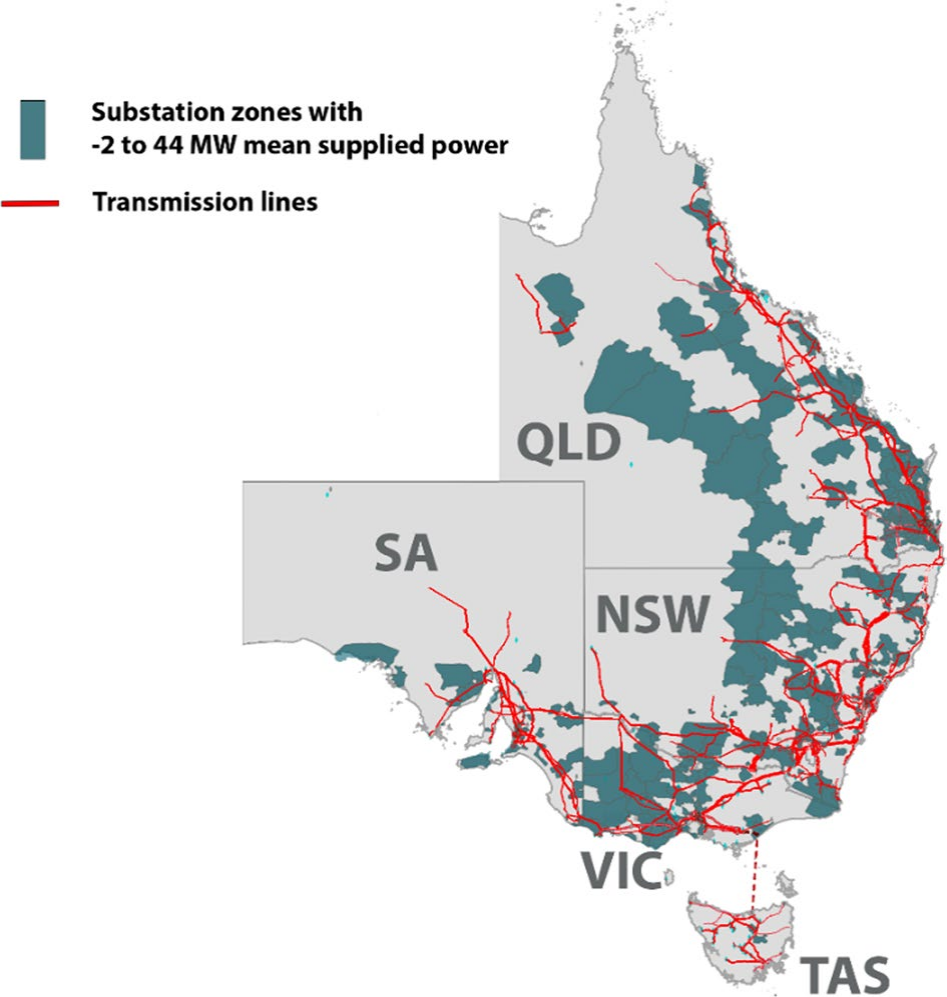
NEM regions, major electricity transmission lines and substation zones (data provided by National Map 2022)

The NEM consists of five regions; New South Wales (NSW), Queensland (QLD), South Australia (SA), Tasmania (TAS) and Victoria (VIC), connected via six interconnector routes. The network regulator, AEMO, provides real-time interconnector and demand power data with half-hourly resolution as well as a full list of participant generators and their SCADA data on its website (AEMO 2022a). Figure 2 shows the NEM’s regions, major electricity transmission lines and substation zones. Here we fetch, process and store these data streams on a cloud server (CSIRO 2022) and apply the power-flow tracing algorithm to compute the consumption-based emissions intensity estimates that are then also uploaded to the cloud in real-time.

At each time-step the generators SCADA data is retrieved from AEMO’s database, formatted to remove inconsistencies, and grouped by region and generation technology. Regional load and interconnector flow data are also imported and formatted to obtain the regional colour mix. Occasionally missing data is filled using linear interpolation. After solving Eq. (2) at each half-hourly time-step, the regional colour mix is obtained. At the final step, this matrix is multiplied by a matrix of average carbon intensity factors of the corresponding region-technology pairs, ${CI}_{region-technology}$ obtained from AEMO (2022a). These values only include the emissions from burning fossil fuels for electricity production in the generators and therefore, the emission intensity factors of renewable generators are reported as zero. Although there are emissions associated with other operational activities and maintenance actions of the generators, to be consistent with AEMO reports and to allow for comparing the results, this research adopted the reported intensity factors by AEMO.

### Battery predictive control module

The amount of power for charging/discharging the battery is modelled using the following discrete dynamic state of charge (SOC) equation:

3 $$SO{C}_{t+1}=SO{C}_{t}+{\eta }_{c}{\left(P{F}_{l}^{PV-Battery}+P{F}_{l}^{Grid-Battery}\right)}\Delta t-\frac{P{F}_{l}^{Battery-Load}+P{F}_{l}^{Battery-Grid}}{{\eta }_{d}}\Delta t-\frac{\gamma }{24}\frac{{E}_{cap}}{100}\Delta t,$$

where ${\eta }_{c}$ and ${\eta }_{d}$ are the charging and discharge efficiency factors (${\eta }_{c}={\eta }_{d}=0.88)$ respectively, $\gamma =0.5\%$ is the self-discharging rate, ${PF}_{l}^{m,n}$ is the power flow from subsystems *m* to *n* at time *l,* and ${E}_{cap}$ is the energy storage capacity of the battery. The selected discretised SOC equation has been adopted from the work of Vahidi et al*.* (2006) and is bounded as follows:

4 $${0.02\le SOC}_{t}\le 0.98.$$

We used a linearised version of the battery degradation model proposed by Schimpe et al*.* (2018) to address the capacity loss due to aging and other operational conditions and to obtain the cost of battery degradation. This semi-empirical degradation model incorporates cycle aging, temperature, current, and state of charge dependent factors for degradation modelling of Lithium-ion Phosphate batteries. The obtained capacity loss from the degradation model at each time-step was converted to a financial cost using a predefined battery specific cost, in $ per kWh, together with estimated fraction of the overall life that the degradation amount represents, assuming no residual value at the end of service-life and an end-of-life capacity of 80% of the starting capacity. This methodology is outlined in detail in Goldsworthy et al. (2022).

PV power generation is estimated using irradiance and weather (temperature and wind speed) data for the site location from the BOM ACCESS-G and ADFD services (BOM 2022) combined with a simplified model of PV module output (Urraca et al. 2018):

5 $${PV}_{gen}\left({Ir}_{eff},{T}_{op}\right)={Ir}_{eff}{\eta }_{rel}\left({Ir}_{eff},{T}_{op}\right){P}_{mod},$$

where ${PV}_{gen}$ is the PV direct current power, ${Ir}_{eff}$ is the effective in-plane irradiance, ${\eta }_{rel}$ is the PV energy conversion factor, ${P}_{mod}$ is the module power, and ${T}_{op}$ is the operational temperature of the module. The operational temperature of a module is usually different than that of the standard testing conditions and this difference should be accounted for when estimating the PV power generation (Urraca et al. 2018):

6 $${T}_{op}={T}_{am}+\frac{{Ir}_{pa}}{{U}_{0}+{U}_{1}{V}_{h}}.$$

In Eq. (6) ${Ir}_{pa}$ is the irradiance on the plane of array, ${T}_{am}$ is the ambient temperature, ${V}_{h}$ is the wind speed at the height of module and ${U}_{0}$ and ${U}_{1}$ are empirical constants. We used the model of Huld et al*.* (2011) to estimate ${\eta }_{rel}$ as follows:

7 $${\eta }_{rel}=1+{a}_{1}\mathrm{ln}{{Ir}_{eff}}^{^{\prime}}+{a}_{2}{\mathrm{ln}}^{2}{{Ir}_{eff}}^{^{\prime}}+{a}_{3}{T}_{op}^{^{\prime}}+{a}_{4}{T}_{op}^{^{\prime}}\mathrm{ln}{{Ir}_{eff}}^{^{\prime}}+{a}_{5}{T}_{op}^{^{\prime}}{\mathrm{ln}}^{2}{{Ir}_{eff}}^{^{\prime}}+{a}_{6}{\left({T}_{mod}^{^{\prime}}\right)}^{2},$$

where ${a}_{i},i=1,\dots ,6$ are regression constants reported by Huld et al*.* (2011), and parameters with a prime are normalised to the standard testing condition. Horizontal irradiance was converted to in-plane irradiance assuming PV modules faced north at a height of 5 m above the ground with a slope equal to the local site latitude (32.928°) and using a ground reflectance factor of 0.5.

### Optimization function

**Fig. 3:**
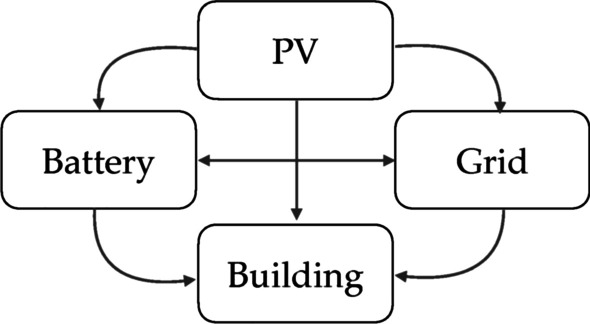
Schematic of the sub-systems and their interactions used in the MPC framework

The schematic of the MPC system designed herein can be found in Fig. 3. The battery is the only controllable sub-system in this MPC framework which was subject to the following energy balance equations:

8 $${BL}_{l}={PF}_{l}^{PV-Load}+{PF}_{l}^{Grid-Load}+{PF}_{l}^{Battery-Load}$$

9 $${PV}_{l}={PF}_{l}^{PV-Battery}+{PF}_{l}^{PV-Grid}+{PF}_{l}^{PV-Load}$$

In Eqs. (8) and (9) ${BL}_{l}$ is the building demand at time $l=1,\dots , t$ where $t$ is the prediction horizon, and ${PV}_{l}$ is the PV generation at time *l*. The following constraints were used:

10 $$0\le {PF}_{l}^{PV-Battery}+{PF}_{l}^{Grid-Battery}\le \mathrm{max}{PF}_{c},$$

11 $${0\le PF}_{l}^{Battery-Grid}+{PF}_{l}^{Battery-Load}\le \mathrm{max}{PF}_{dc},$$

where $\mathrm{max}{PF}_{c}$ and $\mathrm{max}{PF}_{dc}$ are the maximum charge and maximum discharge power of the battery respectively. Here $\mathrm{max}{PF}_{c}=\mathrm{max}{PF}_{dc}=0.5{E}_{cap}$.

The MPC was used to minimise the following multi-objective cost function:

12 $$\sum_{l=1}^{t }\left[{EwCost}_{l}\left({PF}_{l}^{Grid-Battery}+{PF}_{l}^{Grid-Load}\right)+{EdCost}_{l}\left({PF}_{l}^{Grid-Battery}+{PF}_{l}^{Grid-Load}\right)+BCost{Bw}_{l}+{CCost}_{l}\sum_{tech}{Q}_{NSW,tech}{CI}_{NSW,tech}\left({PF}_{l}^{Grid-Battery}+{PF}_{l}^{Grid-Load}\right)\right],$$

where $EwCost$ is either the TOU or wholesale tariff costs (according to the scenario considered), $EdCost$ is the demand exceedance cost applied in the TOU cost scenario, $CCost$ is the carbon price, $BCost$ is the battery degradation cost, $Bw$ is the battery wear to be obtained from a battery degradation model and ${Q}_{NSW,tech}$ and ${CI}_{NSW,tech}$ are the network colour and emissions intensity matrices from the carbon emission model. The demand exceedance cost applies a large cost penalty if the net imported power during demand charging times (workdays between 2 and 8 pm) goes above the current maximum power over any 30 min period in the month. The penalty is applied as a squared value so that large exceedances are penalised more than small exceedances following the methodology of Goldsworthy et al. (2022). This term means that Eq. (12) is non-linear. Here we solve Eqs. (10–12) using the MATLAB^Ⓡ^ QUADPROG function (MathWorks 2022), based on the method described in Kouzoupis et al*.* (2018).

## Emissions intensity on the national electricity market

### Regional and time of day variations

**Fig. 4:**
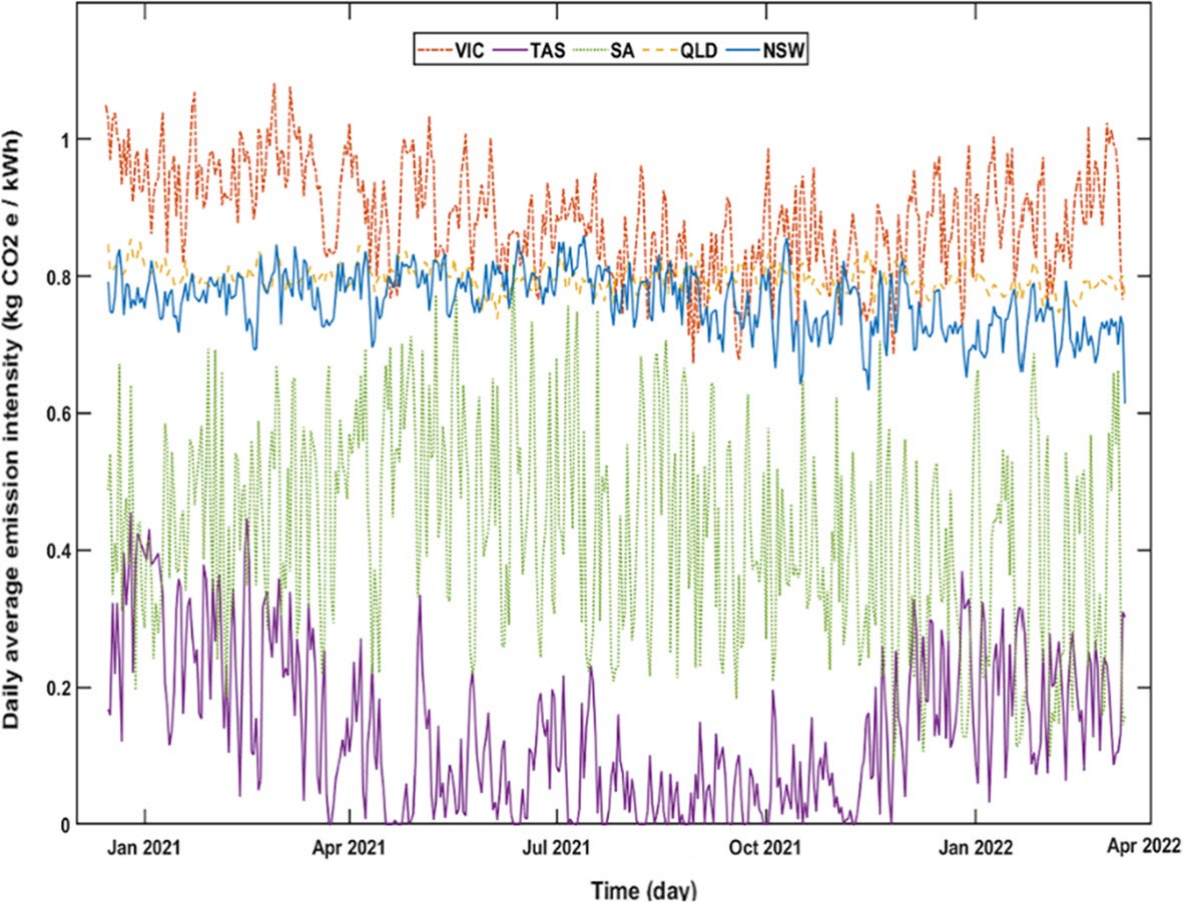
Daily average emission intensity of NEM regions between January 2021 and March 2022

The daily average emission intensities of the five NEM regions for 2021 and 2022 are shown in Fig. 4. VIC has the highest average daily carbon intensity with 0.88 kgCO_2_/kWh followed by QLD (0.79), NSW (0.76), SA (0.43) and TAS (0.13). The overall higher emission intensity in VIC is due to the main source of electricity production in the state, which is brown coal. Compared with black coal which supplies 79% of the electricity production in NSW and QLD, brown coal is more emission-intensive due to its lower carbon content (AEMO 2022c; Jotzo and Mazouz 2015). The carbon intensity factor of the NEM’s brown coal-based generators is between 24.7% and 40% higher than the black coal-based generators (insight gained from data in AEMO (2022a).

Part of VIC’s brown-coal dominant production also contributes to the relatively higher overall emission intensities of SA and TAS compared with the reported numbers from the production-based emission models (Map 2022). Greater inter-day variation occurs for SA and TAS. Production in SA and TAS is 60% and 99% renewable respectively, while the larger fluctuations are a consequence of the fluctuating imports from brown-coal dominated VIC (their only connection point).

**Fig. 5:**
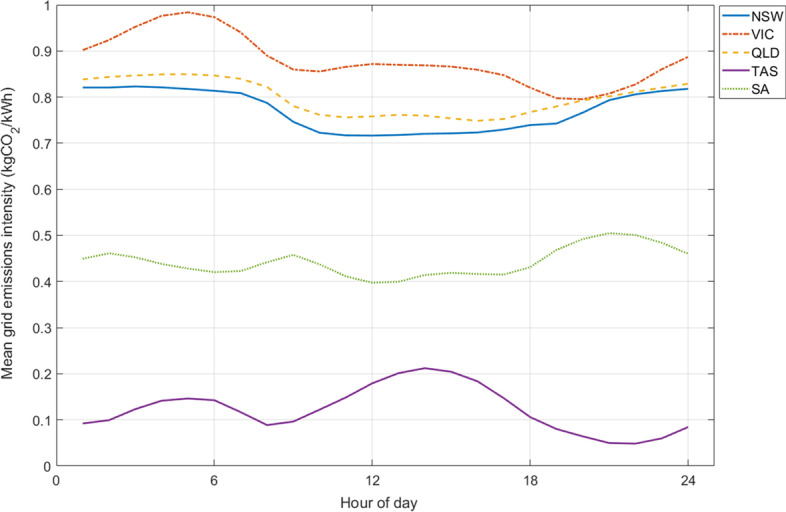
Mean hourly variation of grid emission intensity for the five NEM regions

Figure 5 shows the variation of mean daily emissions intensity by region. The trends for QLD, NSW and SA are similar except that for SA emissions don’t display an increase between midnight and 7:00 AM. VIC’s general trend is similar to TAS’s except with a less pronounced rise over the day peaking in early afternoon, and a more pronounced increase in the early morning. The former is due to a larger portion of solar electricity generation in VIC which has more operating solar farms and rooftop solar panels, and higher average irradiance (AEMO 2022c; Solargis 2022). The same reason justifies the flatter trend for QLD, NSW, and SA (each with 6% annual utility solar contribution) as opposed to the VIC and TAS (with 3% and 0% annual utility solar contribution, respectively).

### Electricity price and emissions intensity correlation

Scatter plots of the half-hourly emission intensity versus the half-hourly electricity wholesale (spot) price are shown in Fig. 11 (see Appendix). Lighter shading indicates more frequent occurrences. In VIC, the highest emissions factors (~ 1.25 kgCO_2_/kWh) occur at intermediate spot prices (between 20 and 50 $/MWh), while the emission intensities for very high spot prices are generally less than 0.8 kgCO_2_/kWh. The overall trend for QLD is similar to NSW, where the highest emissions factors (~ 0.95 kgCO_2_/kWh) occur over a range of spot prices. In general, the emissions intensity factors are low (~ 0.7 kgCO_2_/kWh) when the spot price is less than $20/MWh or higher than $300/MWh. In TAS, the highest emission intensities occur when spot prices are low.

Overall, no strong linear correlation between the emission intensity and the wholesale price has been observed in all the studied regions. To study the dependence (further to monotonic associations), a non-linear correlation parameter (Hoeffding D) was computed and is given in Table 1. This value generally ranges between 0 and 1 with values close to zero indicating no correlation. These results indicate no dependence between the wholesale price and emission intensity for NSW, VIC and QLD and a very small dependence for SA and TAS. This means that the wholesale electricity price signal does not currently drive consumers toward using energy at times when the emissions intensity is low.

**Table 1:** Non-linear correlation between spot price and grid emissions intensity

State	Hoeffding D
NSW	0.0216
VIC	0.0140
QLD	0.0125
TAS	0.1012
SA	0.1130

**Fig. 6:**
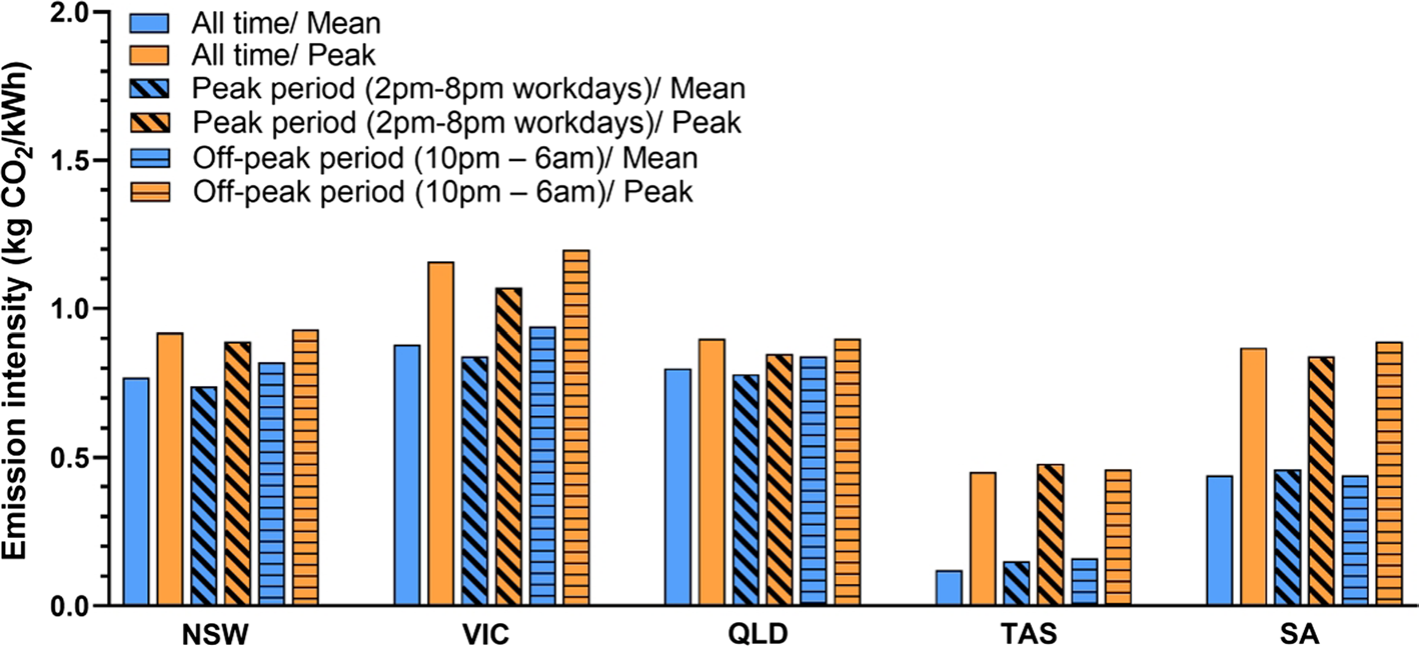
Mean and peak emissions intensity during typical peak and off-peak electricity tariff periods for NEM regions

Most electricity customers are not directly exposed to the spot market price, commonly they are charged pre-determined rates based on the quantity and time of energy use. The mean and peak emission intensities of each region over typical peak and off-peak electricity charging periods is shown in Fig. 6. Except for SA, the mean emission intensities of all the regions during the peak period are lower than during the off-peak period. In TAS, the maximum emission intensity during the off-peak period (0.48 kgCO_2_/kWh) is slightly higher than the maximum intensity during the peak period (0.46 kgCO_2_/kWh). This behaviour is unique to TAS. In general, the existing TOU price signals largely actually drive consumers toward using energy during times when grid emissions intensity is *higher*.

### Operational demand and emissions intensity correlation

The contribution of different generators to overall demand and emissions intensity is also of interest since it provides insight into the scheduling of different types of generators. A cross-correlation analysis of the emission intensity and operational demand, resulted in Pearson correlation coefficient of approximately −0.4 for all the studied regions, except SA. That is, emissions tend to be lower at times of higher operational demand. For SA no statistically significant correlation was observed.

The correlation between specific emissions and operational demand can be related to the unique energy mix of each region, and/or merit order of generation in which cost of operation plays a major role. Open and combined-cycle gas turbines, which tend to have higher running costs, are typically employed during periods of higher operational demand. The emission intensity of these generators is, however, much lower than brown, and black coal-based generators that usually meet the majority of demand during low to moderate demand periods (Elliston et al. 2016; Nelson 2018). The correlation between emission intensity and the operational demand is much less in SA which has a high contribution of both renewables and gas-based generators.

**Fig. 7:**
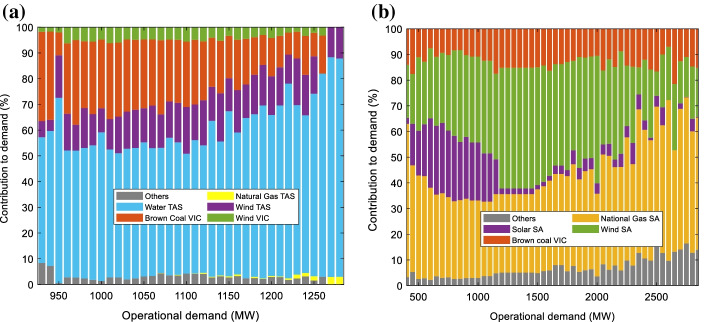
Contributions of the different types of generators to operational demand in **a** TAS and **b** SA

Figure 7 shows the contribution of the major generator types to operational demand in TAS and SA for the first half of 2021–2022. TAS is only connected to VIC and has only one operating non-renewable plant (Bell Bay Three). Therefore, it is relatively easy to predict the emissions behaviour of this region. When operational demand is low, the imported brown-coal-based electricity from VIC is one of the main contributors to the local demand and consequently emissions intensity is expected to be high. In periods of moderate operational demand more local natural gas generated from Bell Bay Three plant is consumed and so emissions intensity is expected to increase. Finally, at times of high operational demand more hydroelectric generation is used and emission intensity is expected to decrease.

For SA (Fig. 7b), 40% of demand was met by local gas-based generators between 2021 and 2022 as opposed to 1% for TAS. At low operational demand, there is a significant portion of local solar generation which tends to be offset by wind generation as demand increases. The high operational demand periods (00:00–6:30 and 19:00–23:30) occur when there is limited output from rooftop and large scale PV systems (AEMO 2022b). In contrast, the abundance of solar generation occurs midday when the operational demand is minimal. This explains the abrupt drop in the contribution of solar to the operational demand in Fig. 7b. For periods of high operational demand natural gas provides a higher fraction of demand but this is offset by a smaller reduction in imported brown coal which results in the emissions intensity being relatively constant.

**Fig. 8:**
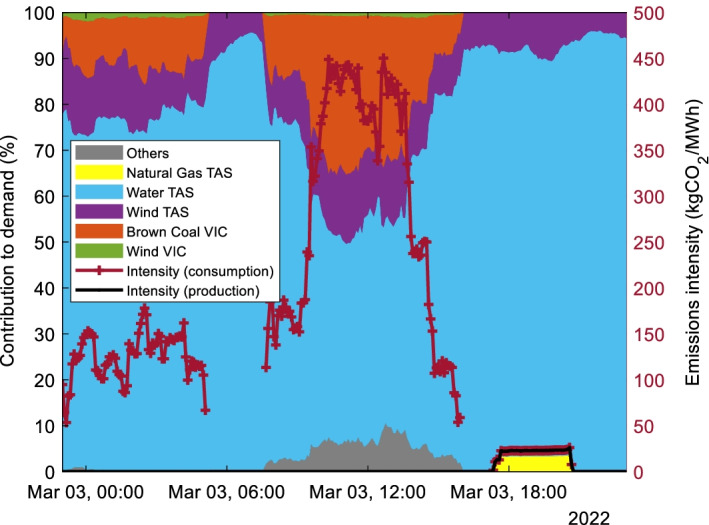
Contribution of different generators to operational demand and consumption and production-based emissions intensity for TAS on 3^rd^ March 2022

The difference between the production and consumption-based emission intensities is also revealing. Figure 8 shows the contribution of the main generators to the demand in TAS on 3rd of March 2022 as well as the production and consumption based TAS emissions intensity. When there is no power imported from VIC and the Bell Bay Three gas generator is operating (5:30–9:30 pm) the consumption and production-based emissions intensity values are the same at 25 kgCO_2_/MWh. However, when the state imports approximately 35% of power from VIC (10 am to 1 pm), the consumption-based emissions intensity increases to 450 kgCO_2_/MWh while the production-based emissions intensity remains zero. This represents an 18 × increased in the daily maximum emissions intensity.

## Commercial building case study

### Overview

The above results indicate that both time of use and wholesale electricity price signals do not necessarily correlate with the emissions intensity of grid consumed electricity. Hence, a trade-off likely existing between designing and managing demand side flexible loads and generators to minimise costs versus emissions. This section explores this trade-off for the case of PV-battery systems applied to a commercial site. The selected case-study is a commercial office and research laboratory site located in Newcastle, NSW, Australia. Pre the COVID-19 pandemic, the site had a typical office occupancy pattern with most staff on-site between 8am to 5 pm M-F. During the 2021 calendar year considered, occupancy patterns were similar to the long-term trend but with total site occupancy reduced by approximately 50%, with the exception of September and October where the site had a nominal occupancy only. Analysis of occupancy patterns from November 2021 until the time of writing (April 2022) reveals that the total site occupancy has not returned to pre-pandemic levels. For this reason, data from the 2021 calendar year was chosen as being representative of the ‘new-normal’ site operation.

The site already generates 30–40% of its energy from existing PV arrays. It also has an existing commercial lithium-ion battery. Here we assess the potential for further minimisation of electricity and carbon emissions from the site through *additional* PV generation and battery storage. That is, the existing PV and battery system is treated as part of the baseline site operation for the purposes of this analysis.

**Fig. 9:**
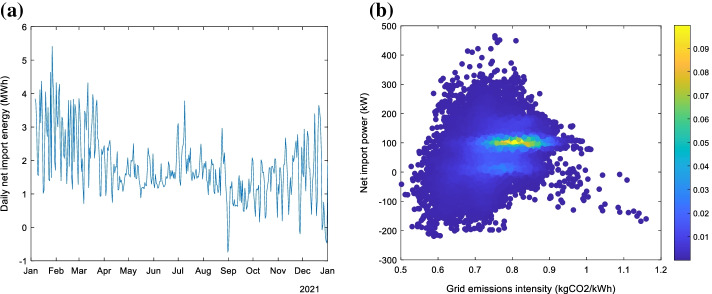
**a** Daily net import energy and **b** scatter plot of net import power vs grid emission intensity coloured by frequency for the case study site.

Historical electricity net import power data at 5-min resolution between January 2021 and December 2021 was obtained. To match the emission model output, the consumption time-series data was oversampled to a 30-min resolution and linear interpolation was used to fill missing time-steps (less than 1.1% of the data). The average daily net import energy is shown in Fig. 9a. Net import was generally higher during the warmer months (particularly January to March), though there is considerable variation which is partially due to changes in site operations. The average daily net import was 1.8 MWh. The existing on-site generation does result in net export of power to the grid at certain times, although this is not apparent from the daily averages.

Figure 9b show a scatter plot of the half-hourly net import power vs the emissions intensity of the grid, with shading indicating the relative frequency of the points (computed using a bi-variate kernel smoothing approach). Net export (i.e. negative import values) to the grid is apparent. It also shows that there are times of very high emissions intensity but that these are infrequent and tend to occur when net import is low or negative. By far the most frequent behaviour is for the net import power to be approximately 90–110 kW which corresponds to the after-hours baseline site power demand, with grid emissions intensity ranging from approximately 0.7–0.9 kgCO_2_/kWh.

### Simulation financial parameters

Simulation financial parameters are summarised in Table 2. The Australian Carbon Credit Unit (ACCU) price is the de-factor price of carbon in Australia and is updated daily through the national carbon market regulated by the Clean Energy Regulator. As of the 9th of February 2022 the price was $55.4 per CO_2_ tonne (Reputex 2022). To study the trade-off between placing more weight on minimising costs versus emissions, carbon prices in the range of $0 to $400/tCO_2_ were considered. That is, when a zero-carbon price is used in the optimisation, the resulting battery charging solution ignores emissions. When a very high carbon price is used, the optimiser places a high importance on minimising emissions, though it still tries to minimise network (TOU) or wholesale costs (according to the scenario considered). Note though that when reporting the effect of changing the optimisation strategy on the annual operating cost the carbon cost was excluded to ensure a fair comparison.

**Table 2:** Case study electricity, capital, and carbon costs values

Parameter	Value/range	References
Battery specific cost	$800/kWh	Guinot et al. (2015) Wood et al. (2015)
PV specific cost	$1000/kW	Helwig and Ahfock (2015)
Network tariffs	Peak periods 2:00–8:00PM workdays off-peak period 10:00 PM-6:00 AM Demand charge during peak period	Energy retailer
Wholesale electricity price	NSW Regional Reference Price	AEMO
Carbon price	$0 to $400 /tonne CO_2_	N/A

We use two different electricity pricing structures: (i) a network (TOU) tariff consisting of a daily fixed charge, TOU-based energy charges and a demand charge; and (ii) a tariff based solely on the wholesale regional reference electricity price (AEMO 2022a). For the network tariff, the TOU charges were applied based on the time of day and type of day. The demand charge applied to the highest net import power over half-hour periods within the peak TOU period over the current month. The network tariffs used are typical of a medium scale commercial site retail tariff. In this case there was no credit for energy exported to the grid. For the wholesale electricity tariff however, any export of power to the grid was assumed to be credited at the  full wholesale electricity price.

The capital costs associated with installation of new batteries and PV arrays were adopted from the literature and are representative of typical battery and PV installations in Australia. Finally, the payback period was calculated here using a net present value approach with a 3% Consumer Price Index (CPI), 5% interest rate and annual maintenance costs of 2% of the annual operating cost.

## Results

The trade-off between controlling the PV-battery system to minimise operating costs versus controlling to minimise greenhouse gas emissions was simulated by varying the carbon price used in the optimisation algorithm. To study the sensitivity to PV-battery system size, a range of different battery capacities (0, 130 kWh, 380 kWh and 640 kWh) and PV array sizes (0 kW, 150 kW, 250 kW and 560 kW) were considered.

**Fig. 10:**
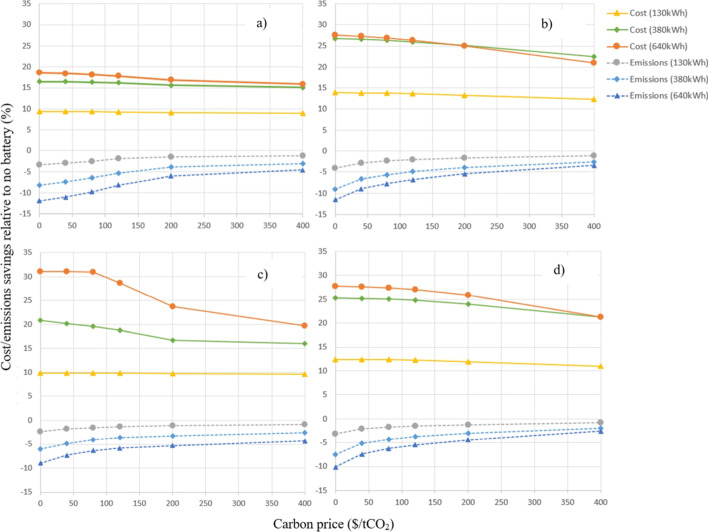
Sensitivity of relative annual operational costs and emissions saving to the weighting factor used by the optimiser for minimising emissions (i.e. carbon price) for; **a** network tariff scenario and 150 kW PV array, **b** wholesale tariff scenario and 150 kW PV

The annual operational cost (*excluding* the carbon cost) and the annual emissions are shown in Fig. 10 as functions of the carbon price. Higher carbon price corresponds to the optimiser placing more weight on minimising emissions. The top two figures correspond to the small (150 kW) PV array, the bottom two to the large (560 kW) PV array. The left and right figures are the network and wholesale tariff scenarios, respectively. Costs and emissions are normalised by those for the case with 0kWh battery and the same PV array size.

**Fig. 11:**
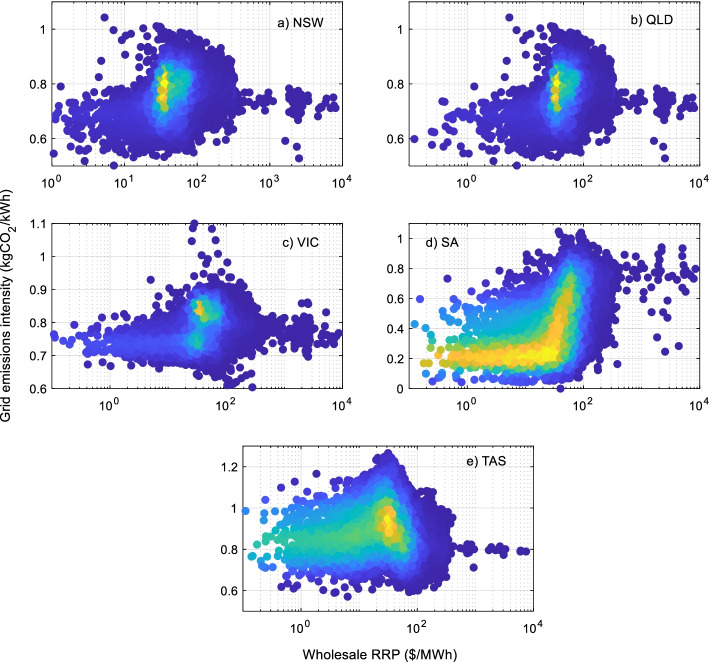
Scatter plot of grid emissions intensity vs the wholesale (spot) price for NEM regions. **a** NSW. **b** QLD. **c** VIC, **d** SA. **e** TAS. Shading indicates frequency of occurrence

For all cases, the addition of the battery storage system leads to operating cost savings but an increase in overall emissions. This occurs because the opportunity for minimising costs (i.e. the variation in tariffs with time) is much greater than for emissions. Using the battery results in an overall increase in energy use due to system losses, and the emissions reduction caused by shifting grid import to times of lower emissions intensity is not enough to offset this in this case.

Figure 10a shows that for the largest battery capacity and a 150 kW PV array, controlling the battery to minimise costs under a zero carbon price results in an 18% saving in operating cost and a 12.5% *increase* in emissions (compared to the 150 kW PV system with no battery). Changing the optimisation strategy to favour reduced emissions decreases the cost saving to 16% but lowers the increase in emissions to 5%. For small battery capacity the magnitude of the effects is reduced because there is less potential to optimise either costs or emissions. For a larger PV array size, the magnitude of the changes is increased because excess PV generation is available to charge the battery, and while this excess is assumed to reduce emissions via a carbon credit, it doesn’t reduce costs given the absence of a feed-in electricity credit.

Figure 10c shows that for the 560 kW PV array and largest battery capacity, the cost saving decreases from 31 to 20% and the emissions increase changes from 9.5% to 4.5% (relative to the 560 kW PV array with no battery). The comparatively higher cost saving at lower carbon price for the 380 kWh and 640 kWh batteries is due to demand charge savings that are lost as the battery is used increasingly for minimising emissions (i.e. for higher carbon prices) where it has less availability to reduce the demand charge.

For the wholesale cost scenarios (Fig. 10b, d) relative cost savings are greater while relative emissions savings are almost identical. This occurs because there is more opportunity for cost arbitrage with the wholesale prices which have a greater variation over a shorter period of time while the potential for carbon emissions-based arbitrage remains the same.

The trends shown in Fig. 10 are normalised relative to the same size PV system (i.e. either 150 kW or 560 kW) but without a battery. Evaluating savings relative to the case without any PV or battery system reveals that the PV system contributes a large portion of the overall cost savings and significantly reduces emissions. For example, under the network tariff the 150 kW, 640 kWh battery system delivered 30% cost and 39% emissions savings relative to the system without any PV or battery when cost minimisation was the only objective. This compares to 27% and 43% for the highest carbon price case. This highlights the fact that in general, lowering grid import power to reduce costs through onsite PV generation has a clear commensurate benefit in terms of reducing emissions, and that the variation in grid emissions intensity for NSW does not provide a large opportunity to use a battery for carbon emissions-based arbitrage.

Studying the overall payback period for both the tariff scenarios shows that payback is largely determined by PV-battery size with larger PV lowering payback and larger battery increasing payback. In general, the variation in payback period with carbon price follows similar trends to the variation in operating cost. Considering the network tariff scenario, for the 640kWh battery capacity, varying the PV size from 250 to 0 kW resulted in the payback period increasing from 21 to more than 50 years. For a 250 kW PV array, varying the battery capacity from 0 to 640kWh resulted in the payback increasing from 12.5 to 21 years. For the wholesale tariff, the payback periods were much longer since the overall operational costs were much lower (approximately half). In practice, wholesale-based tariffs are likely to include other charges that will affect this payback.

Finally, the influence of possible future reductions in PV and battery capital costs was considered. PV costs was assumed to be $700/kW and battery cost $300/kWh. For the 130kWh, 150 kW PV-battery system, the resulting payback period reduced by 45% (network tariff) and 56% (wholesale tariff). However, these changes to capital costs did not affect the operating cost-emissions trade-off.

### Limitations and recommendations for further research

The case study analysis based on NSW prices and emissions found that controlling a battery to minimise either the network tariff or the current wholesale spot price did not lead directly to minimisation of emissions. Analysis of the variation of grid emissions intensity over different times of the day and the correlation with the wholesale spot price indicates that similar results would be likely for VIC and QLD. Future research could include case study analyses for the other NEM regions, particularly SA and TAS.

This research did not consider the time-variable nature of the carbon price including a dynamic rather than fixed carbon price may influence the results particularly if the price fluctuations occur over timescales where battery-based arbitrage is feasible. Expanding the scope of ‘emissions’ to account for the embodied emissions contained within the distributed energy systems and also the indirect emissions associated with renewable energy generators would be a worthwhile topic of investigation. Finally, as the emissions intensity factor of the grid is gradually reducing over time, it is suggested to revisit the analysis in future years.

The assumption of a perfect emission intensity forecast should also be investigated since it will influence the extent to which theoretical savings can be achieved in practice. Additional considerations for practical deployments include handling data quality including data drop-outs, equipment and communications reliability issues and security, for example as discussed by Taylor et al. (2019). The time-series format of the emission intensity factors from the emission model allows for forecasting the emission intensities using different time-series prediction methods. Therefore, it would be possible to feed the forecast emission intensity data into the real-time demand response frameworks for real-time optimisation and control. Such a model could also consider uncertainty ranges associated with the emissions intensity forecasts, for example using information on the variability of generation mix, or more generic time-series uncertainty approaches.

This analysis used the calculated consumption-based grid emissions factors to optimise costs and emission for a specific end-use application (i.e., a commercial site). An alternative analysis could consider the inverse problem of optimising the generation mix to achieve certain network level emissions targets while minimising overall costs. Expanding this further, it would be useful to develop a digital twin emissions model for the NEM. Such a model could be used, for example, in what-if scenario analysis to assess the cost, robustness, performance, and emissions reduction of novel energy projects. Finally, consideration of how time-dependent grid emissions intensity could be factored into network tariffs and the wholesale spot price would enable cost optimal and emissions optimal outcomes to be more closely aligned.

## Discussion and conclusion

This primary goal of this research was to investigate the extent to which a PV-battery system controlled to minimise electricity costs also results in carbon emissions savings (and vice-versa). This was simulated by varying the carbon price over a wide range and using the MPC framework to minimise the sum of the electricity bill cost and the cost associated with the emissions from consumption of grid electricity at the assumed carbon price for case study commercial site. That is, the carbon price was used as a weighting factor to incorporate the emission optimisation into the MPC framework. Two different tariff structures were considered. To account for the grid emissions at the site’s location a real-time consumption-based model of the NEM was first developed. The developed emissions model was to analyse how the emission intensity of the NEM varies over time, operational demand and with the wholesale spot price. Major findings were:

- The general emissions intensity behaviour of the different regions varies over the day and this variation was associated with that region’s ratio of renewable to non-renewable generation as well as its major source of local productions.
- Consumption and production-based emissions estimates vary considerably. While VIC had both the highest average consumption-based and production-based emissions intensity within the studied period, TAS and SA’s consumption-based emissions were found to be much higher than their production-based emissions. This has important implications for calculation of emissions, and, for example for measuring progress toward NZE for sites consuming grid purchased electricity.
- The emission intensity was found to be correlated with the TOU prices. However, the existing TOU price signals were found to encourage consumers to use energy during times when grid emissions intensity is *higher*, rather than when it is lower. This is mostly due to higher TOU price periods being in the evenings when renewable generation is less. In the case of wholesale prices, there was generally no correlation between the spot price and the emissions intensity.

The key findings from the case study analysis were:

- Controlling a battery to minimise operating costs comes at the expense of increasing emissions. This occurs for both the network (TOU) and wholesale tariff scenarios and is not affected by capital costs.
- Whereas PV arrays directly reduce both costs and emissions by reducing use of grid electricity, the ability of batteries to reduce cost and emissions is contingent on the time variation of the network or wholesale tariffs and the time variation of the grid emission factor. These variations must be large enough to overcome the increased overall energy use that is associated with batteries (due to charge/discharge inefficiencies) before any savings can be realised. Here cost savings were apparent but not emissions reductions.
- There is a much greater potential to use a battery to reduce costs for larger PV arrays with regular net export under network tariffs where there is no feed-in credit. However, the assumption of a carbon credit for emissions reduction due to exported power means that a similar increased potential to use the battery to reduce emissions for large PV arrays was not apparent.

Overall, the analysis shows that there is potential for existing common tariff structures to be modified to provide greater incentive for consumers to shift grid electricity consumption to times when grid emissions intensity is lower. In the short to medium term while the overall load shifting capacity from distributed consumers remains small, there is unlikely to be significant feedback effect from this load shifting on either the price signals or the grid emissions intensity factors. Hence it is reasonable to consider them as decoupled. In the long-term the influence of fluctuating price signals due to large scale demand shifting will need to be considered. Ultimately the benefit will be to increase the value of low emissions generation specifically at times where it can have the most emissions reduction benefit.

## Data Availability

The AEMO data-sets used in this study are available from the supplied references. The calculated electricity network consumption-based emissions intensity data-set and the commercial building baseline power consumption data-set are available from the corresponding author upon request.

## Appendix

See Fig. 11.

## Abbreviations

ACCU: Australian Carbon Credit Unit
AEMO: Australia Energy Market Operator
BOM: Bureau of Meteorology
GHG: Greenhouse gas
QLD: Queensland
MEF: Marginal emissions intensity factor
MPC: Model predictive control
NEM: National Electricity Market
NSW: New South Wales
NZE: Net zero emissions
SA: South Australia
SCADA: Supervisory control and data acquisition system
TAS: Tasmania
TOU: Time of use
VIC: Victoria
